# The Safety and Efficacy of Posterior Lumbar Interbody Fusions in the Outpatient Setting

**DOI:** 10.7759/cureus.53662

**Published:** 2024-02-05

**Authors:** Hunter F Pharis, Daniel T DeGenova, Braden J Passias, Taylor J Manes, Grace Parizek, Daryl Sybert

**Affiliations:** 1 Orthopedic Surgery, OhioHealth System, Columbus, USA; 2 Orthopedic Surgery, Ohio University Heritage College of Osteopathic Medicine, Columbus, USA; 3 Orthopedic Surgery, Mount Carmel Health System, Columbus, USA

**Keywords:** open surgery, spinal fusion, same-day discharge, outpatient lumbar fusion, posterior lumbar interbody fusion

## Abstract

Introduction

Outpatient surgical procedures have shown reduced costs, improved patient outcomes, and decreased postoperative complications. Interest in moving orthopedic and neurosurgical spine procedures to the outpatient setting has grown in recent years because of these factors. Studies investigating open posterior lumbar interbody fusions (PLIFs) in the outpatient setting are sparse.

Methods

The patients who underwent an open PLIF with pedicle screw and rod construct from 2014 to 2018 were retrospectively reviewed. Outpatient procedures were defined by patient discharge being on the same day of the procedure, without admittance to an inpatient ward. Pertinent demographic, clinical, radiographic, and surgical data were collected and analyzed.

Results

The current study included 36 outpatient PLIF cases with 94.4% of the study cohort undergoing a single-level PLIF. The average Oswestry Disability Index (ODI) score improved by 20.4 points from preoperative measurements (p = 0.0002), and the visual analog scale (VAS) score improved by 27.2 points (p = 0.0001). The postoperative fusion rate was 94.4%. One intraoperative complication occurred (2.78%), and four postoperative complications occurred (11.11%). There were no subsequent admissions throughout the postoperative follow-up period; however, two of the 36 patients (5.56%) did require reoperation, both in an outpatient setting.

Conclusions

This study demonstrates that open posterior lumbar interbody fusions performed in an outpatient setting can be performed safely and effectively, with a significant reduction in VAS and ODI pain scores.

## Introduction

Throughout the last two decades, the rates of spine surgeries have seen consistent growth in both the inpatient and outpatient settings [1,2]. Specifically, posterior lumbar interbody fusions (PLIFs) have become more common as new instrumentation has developed and procedural techniques have improved [3]. The number of spinal fusions has increased by 62.3% from 2004 to 2015, representing a 177% increase in spending [1]. In an effort to reduce the overall cost of spinal surgery, surgeons are choosing to perform procedures in a setting where it can be done both safely and effectively. Outpatient procedures have shown several benefits, including the reduction of associated healthcare expenses, decreased exposure to infections, and reduced risk of medical errors [4,5].

Due to these benefits, the number of outpatient PLIFs performed has continued to rise; however, the data on outpatient open PLIF remains sparse and without consensus. Outpatient open posterolateral single-level spinal fusions have demonstrated a significant decrease in visual analog scale (VAS) and Oswestry Disability Index (ODI) scores, as well as a high fusion rate with a low incidence of complications [6]. Similarly, a recent study demonstrated comparable complication rates between the patients undergoing inpatient and outpatient posterior lumbar fusions [7]. In the cervical spine, Boddapati et al. state that patient selection is the most important factor when determining whether outpatient surgery is offered for three- and four-level anterior cervical discectomies and fusion procedures [8].

The safety and efficacy of outpatient open PLIFs have not been thoroughly investigated. In the past, this has largely been due to the limitation of outpatient surgery sample size. This study is a retrospective report on the clinical and radiographic outcomes of 36 patients who underwent outpatient open PLIFs in a single surgery center by two orthopedic spine surgeons.

## Materials and methods

Study design

This was a retrospective case series.

Inclusion and Exclusion Criteria

A retrospective review of all patients undergoing open outpatient posterior lumbar interbody fusions (PLIFs) from January 1, 2014, to December 31, 2018, at a single institution was conducted. The inclusion criteria consisted of all patients, aged 18-85, who have undergone either a one- or two-level outpatient open PLIF between the dates of January 1, 2014, and December 31, 2018. Each patient underwent decompression with posterior interbody insertion and pedicle screw and rod constructs with a posterolateral fusion. PLIF indications included those with degenerative disc disease, spinal stenosis, spinal instability, recurrent disc herniation, and sciatica. There were no trauma patients included in this study. The exclusion criteria included patients with spinal tumors, previous spinal infections, and greater than a two-level open PLIF.

Ethical Approval

Prior to data collection, we obtained ethical approval from the Mount Carmel Health System Institutional Review Board, Columbus, Ohio (approval number: 200430-11). The study adhered to the rules and regulations listed by the review board regarding human research.

Technique

A midline incision was made over the pathological spinal elements. Subperiosteal dissection was completed and followed by the localization of respective lumbar vertebrae using intraoperative fluoroscopy. Instrumentation is then followed with bilateral pedicle screw placement in a standard fashion with a burr, blunt-tipped awl, ball tip feeler, and appropriate taps. Decompression then followed with the removal of the midline elements. The bone was saved and denuded of soft tissue for subsequent morselization and autografting in later steps. Following posterior spinal decompression with the removal of spinous processes, lamina, medial facets, and corresponding ligamentum flavum, attention was then directed toward interbody fusion. The placement of the unilateral cage was performed on the side of the most symptomatic lower extremity with regard to radicular symptoms. To allow for adequate access to the anterior column, further facetectomy was carried out laterally at the respective pathological side.

Facetectomy was performed in a stepwise progression with a series of Kerrison Rongeurs until Kambin's triangle was visualized with the exposure of both the exiting and traversing nerve roots at the level of the intervertebral disc. After adequate exposure to the pathological disc was obtained, a right-angle nerve root retractor was placed at the lateral aspect of the traversing nerve root to retract it medially. The removal of the respective disc was then performed in a standard fashion with an annulotomy knife, disc spreaders, shavers, and a series of curettes and pituitaries. Static interbody cage size selection was determined by manual feedback from disc space spreaders and shavers, as well as preoperative advanced imaging of adjacent disc spaces. Height was chosen to maximize indirect foraminal decompression while also not predisposing to subsidence. The interbody cage was then packed with a morselized autograft and impacted into place. The adequacy of decompression was then assessed with a right-angle nerve hook; this was deemed satisfactory when a 4 mm probe could pass freely through the foramen and the traversing nerve root exhibited 1 cm of medial displacement at the level of the lateral recess. Rods were then placed with posterolateral autografting and allografting (demineralized bone matrix) to promote posterolateral fusion mass. Meticulous hemostasis was obtained prior to closure, and no drains were placed. Closure was obtained in a traditional fashion with absorbable sutures placed at the deep lumbodorsal fascia, subcutaneous tissue, and skin. Postoperatively, the patients were not placed on pharmacologic deep venous thrombosis prophylaxis.

Data Collection

After formal institutional board review approval was obtained, current procedural terminology (CPT) codes were used to survey all patients operated on within the respective time window to delineate those having undergone PLIF in an outpatient setting. The electronic medical record was used to obtain demographic data, surgical data, and postoperative outcome measurements. Oswestry Disability Index (ODI), visual analog scale (VAS), and plain radiographs were obtained at each postoperative visit. Primary outcomes included how many levels were included in the PLIF, levels fused, and the length of hospital stay. Secondary outcomes included drain placement, readmission rates, and postoperative complications including urinary retention, nausea, or vomiting. Readmission was considered for the first 90 days postoperatively.

Statistical Analysis

Statistical analysis was performed, with continuous variables reported as mean values and standard deviations and with categorical variables reported as percentages. Microsoft Excel (Microsoft® Corp., Redmond, WA) was used for data computation.

## Results

A total of 881 patients underwent PLIF surgery between January 1, 2014, and December 31, 2018. Of this total, 845 patients underwent inpatient admission after their operative spinal fusion. This left a total of 36 patients who met the inclusion criteria and underwent a one- or two-level outpatient PLIF. The average age was 48 years, and 58% of the patients were male.

Table 1 provides a summary of the demographic data.

**Table 1:** Demographics of the patients who underwent a posterior lumbar interbody fusion from 2014 to 2018 BMI, body mass index; ASA, American Society of Anesthesiologists; n, number of patients

Variable	Result, n (%)
Age (years)	48.03 ± 9.3 (32-68)
30-42	10 (27.8%)
43-55	17 (47.2%)
56-68	9 (25.0%)
Sex (male)	21 (58.3%)
BMI (kg/m^2^)	30.5 ± 5.4
17-25	6 (16.7%)
25-30	8 (22.2%)
30-35	14 (38.9%)
>35	8 (22.2%)
ASA score	2.02 ± 0.30 (1-3)
1	1 (2.8%)
2	32 (88.9%)
3	3 (8.3%)
Tobacco use (yes)	12 (33.3%)
Hypertension (yes)	9 (25.0%)

Out of the 36 patients, 94.44% (34/36) had a single-level PLIF and 5.56% (2/36) had a two-level PLIF performed. The most common levels instrumented were L5-S1 (61.11%), L4-L5 (33.33%), and L4-S1 (5.56%). The mean length of stay was 6.06 ± 1.2 hours. Only 5.56% (2/36) of the population had a drain placed intraoperatively due to increased blood loss, which was removed by the family on postoperative day 3. No patients in the study suffered from postoperative urinary retention, while three (8.33%) patients suffered from postoperative nausea and vomiting in the early postoperative period.

Table 2 provides a summary of the operative data.

**Table 2:** Operative results of the patients who underwent a posterior lumbar interbody fusion from 2014 to 2018 EBL, estimated blood loss; n, number of patients

Variable	Result, n (%)
Levels fused	
L4-L5	12 (33.3%)
L5-S1	22 (61.1%)
L4-S1	2 (5.6%)
Number of levels fused	1.06 ± 0.23 (1-2)
One level	34 (94.4%)
Two level	2 (5.6%)
Length of stay (hours)	6.06 ± 1.2
40-59	21 (58.3%)
60-79	12 (36.1%)
80-100	2 (5.6%)
Surgical time (minutes)	63.9 ± 12.5
40-59	17 (47.2%)
60-79	14 (38.9%)
80-100	5 (13.9%)
EBL (mL)	181.9 ± 240.0
0-199	26 (72.2%)
200-400	9 (25.0%)
>400	1 (2.78%)

One intraoperative complication occurred (2.78%) within this study population, which was an incidental durotomy that was fixed via primary repair with a 4-0 silk suture in a simple interrupted fashion. Repair is reinforced with an absorbable regeneration matrix. Repair is then tested with an anesthesia Valsalva maneuver to 40 mmHg and held for 10 seconds. The patient was an obese 49-year-old male who underwent a previous laminectomy at the index level. The patient convalesced at the outpatient center and was discharged home in stable condition without any subsequent complications.

The average follow-up was 12.2 months. There were no readmissions or infections in the postoperative period. The conversion rate from outpatient to inpatient was 0%.

Table 3 provides a summary of the postoperative outcome measurements.

**Table 3:** Postoperative outcomes of the patients who underwent a posterior lumbar interbody fusion from 2014 to 2018 ODI, Oswestry Disability Index; VAS, visual analog scale; n, number of patients

Follow-up (months)	Underwent fusion, n (%)	Conversion to inpatient, n (%)	Complications, n (%)	Reoperation, n (%)	Infection, n (%)	ODI score (preoperative)	ODI score (postoperative)	VAS pain score (preoperative)	VAS pain score (postoperative)
12.2 ± 6.2	34 (94.4%)	0 (0%)	4 (11.1%)	2 (5.56%)	0 (0%)	46.6 ± 3.1	26.3 ± 3.8	62.7 ± 4.6	35.5 ± 5.4

The mean ODI preoperative pain score of 46.6 ± 3.1 improved significantly to a mean score of 26.3 ± 3.8 (p = 0.0002) at the final follow-up. The mean VAS lower back pain score of 62.7 ± 4.6 improved to a score of 35.5 ± 5.4 (p = 0.0001) at the final follow-up. The overall fusion rate was 94.4% (34/36), with a 5.56% (2/36) rate of nonunion at one-year follow-up. Eight of the 36 patients did not follow up at one year because they were released to full activity earlier than expected. They were released prior to their one-year follow-up appointment because they were clinically doing well, and the final radiographs demonstrated an uneventful and successful fusion.

There were four (11.1%) postoperative complications in the study population. One was a hardware failure in the setting of a broken pedicle screw. The patient was asymptomatic, and this did not require intervention as the patient went on to a successful union of the fusion mass. A second patient had uncontrollable pain five days postoperatively and presented to the emergency department. She was neurologically intact and discharged from the emergency department with additional oral pain medication. Two patients who underwent a one-level PLIF had symptomatic pseudoarthrosis of their fusion based on advanced postoperative imaging and underwent a revision surgery at the index level. Both went on to successful fusion. None of the patients with postoperative complications had a previous history of smoking. Only 5.56% (2/36) required an additional operation in this study.

## Discussion

The rise in the rates of elective lumbar fusion surgeries in the last 20 years is likely multifactorial in cause and has had a significant economic impact. With 122,679 cases performed in 2004 and 199,140 performed in 2015, lumbar fusion surgery aggregate costs have increased by 177% [1]. During that time, surgeons have increasingly used interbody procedures to increase anterior column fusion mass and promote indirect decompression [9]. A form of indirect decompression that has been studied is longitudinal distraction. Ligamentotaxis provides indirect decompression by distracting the caudal from the cephalad vertebra and reducing the bone fragments from the spinal canal back to the vertebral body [10]. Accompanying these changes, fusion rates and patient outcomes have improved [9]. Even with the advancements of new techniques, the traditional posterior approach to fusion is still the technique most often used by surgeons. Many spine surgeons are well trained and extensively experienced in this procedure. The single midline incision allows for the excellent visualization of the nerve roots, with 360-degree fusion and anterior column support [11].

Despite the recent improvements in the field of lumbar surgery, certain challenges still remain. One such challenge is the cost of inpatient fusion surgeries. A study by Martin et al. reported average costs of over $50,000 for inpatient lumbar fusion procedures [1]. A review article by Mikhail et al. found that outpatient short-segment lumbar fusion can have significant cost savings of up to 65%-70%, mainly by reducing admissions [12]. These findings were supported by a retrospective cohort study of 100 patients comparing the postoperative course for both inpatient and outpatient spinal fusions, with a significantly reduced economic burden for those in the outpatient group [13]. Of note, selection bias is likely present in the patients who receive outpatient fusions. A meta-analysis of 370,195 patients with outpatient spinal surgeries found that while they did have better short-term outcomes and reduced costs, a significant selection bias was present due to confounding variables such as younger patient age and decreased comorbidities [2].

In our report of patients undergoing outpatient open PLIF, solid fusion was demonstrated both clinically and radiologically in 94.44% of the patients. We report a low complication and reoperation rate, as well as an improvement in clinical outcome scores. The patients reported overall positive outcomes with a statistically significant decrease in ODI scores (p = 0.0001) and VAS scores (p = 0.0002).

A study by Asher et al. examined the threshold for the minimum clinically important difference (MCID) in ODI scores in the patients who underwent lumbar spine surgery at one year of follow-up [14]. The MCID in ODI scores ranged from 3.3 to 26.6, depending on the metric used, and they determined the MCID to be a change of 14.3 points in the ODI score [14]. Our study reports a decrease in mean ODI scores of 20.4 points, which points to a clinically important difference. A meta-analysis of 37 studies reported the MCID in pain scores such as VAS and the numeric rating scale [15]. The MCID for VAS ranged from 8 to 40 in various studies, with a median of 17 [15]. The 27.2-point decrease in VAS reported in our study further illustrates that the improved postoperative outcomes are of clinical significance.

Patient outcomes measured by complications appear to point to the safety of outpatient open PLIFs in this study. A single intraoperative complication was noted, which was an incidental durotomy. This was a revision surgery, in an obese male, with intraoperative findings of fibrosis. The patient recovered without further complications. Two patients required reoperation one year after surgery due to pseudoarthrosis, neither of whom had any previous history of smoking. The rate of observed pseudoarthrosis in this study was 5.56%, which is similar to previously reported studies [16,17]. Chin et al. reported outcomes of 16 patients receiving outpatient PLIF and transforaminal lumbar interbody fusion (TLIF) surgeries with a fusion rate of 87.5% [6]. Only one postoperative complication was reported in a female patient presenting with aseptic/low-grade discitis after failure to take postoperative oral antibiotics [6]. In a report of 74 PLIF and TLIF surgeries, Asil and Yaldiz reported a dural injury in 12 patients, who all recovered fully without any additional complications [18].

The mean operation time reported in our study was 63.9 ± 12.5 minutes, which is comparatively short for this procedure in the review of recent literature. Surgical time was recorded as time from incision to time of surgical closure. A study of 40 patients by Ntoukas and Müller showed a mean surgical time of 152 minutes in an open PLIF group compared to 275 minutes in a minimally invasive surgery (MIS) group [19]. A study by Cheng et al. found that a reduction in surgical time was linked to a decreased risk of surgical site infection (SSI) [20]. The low complication rate observed in this study may be partially explained by decreased operative time.

SSIs have been thoroughly studied in the literature. Superficial SSI involves the skin and subcutaneous tissues and occurs within 30 days of the procedure [21], whereas deep SSI involves the fascia and muscle and occurs within one year of the procedure [21]. SSI complications may require antibiotics or even a second procedure to treat them. Factors such as renal insufficiency, hemodialysis, autoimmune dysfunction, and glucocorticoids have been linked to infection postoperatively [21]. However, more literature to support these claims is warranted, as well as the pathophysiology behind each risk factor.

One limitation of this study is a short follow-up of one year; however, each patient was doing well at the final follow-up. An additional limitation is the lack of a comparison group to an inpatient cohort. The advantages of this study include a larger patient population compared with similar studies and a homogenous group of patients, all of whom underwent an open PLIF procedure by one of two surgeons. The conclusions regarding the safety and efficacy of this procedure would be bolstered by a larger, prospective randomized controlled trial to assess patient outcomes with minimal bias. Further investigations could detail this procedure in juxtaposition to an inpatient control group, as well as assess the safety and efficacy of outpatient interbody fusions through direct lateral or anterior-to-psoas approaches.

## Conclusions

This retrospective review detailed the clinical outcomes of one- and two-level posterior lumbar interbody fusions performed in an outpatient setting. The clinical outcome data from this patient cohort showed no readmissions, as well as low complication and reoperation rates. In addition to high fusion rates, this sample showed a significant reduction in both ODI and VAS clinical outcome scores. Ultimately, this investigation supports the notion that posterior lumbar interbody fusions are both safe and effective when completed in an outpatient setting.
